# Blood Magnesium levels in migraineurs within and between the headache attacks: a case control study

**Published:** 2012-03-15

**Authors:** Afshin Samaie, Nabiollah Asghari, Raheb Ghorbani, Jafar Arda

**Affiliations:** 1 Semnan University of Medical Siences, Semnan, Iran

**Keywords:** Migraine, Magnesium, Blood, Headache

## Abstract

**Introduction:**

Some probable mechanisms have been described to the relationship between magnesium (Mg) level and migraine headache attacks. In the study reported here, we sought to determine the total Mg serum status of patients with migraine within and between the headache attacks and compare it with non-migraineurs.

**Methods:**

This study was performed on 50 migraineurs patients diagnosed according to the International Headache Society (IHS) criteria for acute migraine headache. Fifty healthy subjects without any family history or evidences of migraine were randomly selected from hospital personnel as the control group. Serum Mg level was measured by Xylidyl blue method.

**Results:**

In the group with migraine headache, no significant difference was found in the serum total Mg levels within and between migraine headache attacks (1.86 ± 0.41 mg/dl versus 1.95 ± 0.35 mg/dl, p = 0.224). But, serum total Mg level was notably lower in the group with these attacks compared to the control group (1.86 ± 0.41 mg/dl versus 2.10 ± 0.23 mg/dl, p < 0.001).

**Conclusion:**

Serum Mg level is on average significantly reduced in patients with migraine compared to the healthy group. However, the serum total Mg levels in migraineurs remained constant within and between migraine headache attacks.

## Introduction

Comparing migraineurs and non-migraineurs suggests a lower low systemic magnesium (Mg) levels in serum, blood cells, saliva, and even cerebrospinal fluid that results in this hypothesis that the migraine headache might be Mg-deficiency disease [1]. Some probable mechanisms have been described to the relationship between Mg level and migraine headache attacks. Of interest is the interaction of Mg with calcium channels, in the light of Mg deficiency in brain cortex of migraineurs that reducing magnesium can result in calcium channels opening, increased intracellular calcium, glutamate release, and increased extracellular potassium, which may in turn trigger cortical spreading depression [2]. Mg can also modulate the level of nitric oxide in the cell, which is deficient in migraineurs [3], especially in women that potentially mediated by female sex hormones [4]. Moreover, low Mg may be associated with increased platelet aggregation, increased serotonin, and, therefore, vasoconstriction that can be important underlying etiology for triggering migraine [5]. In the study reported here and for more emphasize, we sought to determine the total Mg serum status of patients with migraine within and between the headache attacks and compare it with nonmigraineurs.

## Methods

This study was performed on 50 migraineurs patients diagnosed according to the International Headache Society (IHS) criteria for acute migraine headache referred to the emergency ward of the Fatemieh hospital, Semnan. All patients aged more than 18 years, initial age for migraine was less than 50 years old, and at least three months was passed from preventive medications, including ß- blockers, calcium channel blockers, or valporate. All migraineurs were studied during their post-ictal phase and at least one week after their last headache attack. Those with the history of diabetes, hypertension, renal dysfunction, gastrointestinal disorders or malnutrition, or thyroid disorders were excluded. Also, the use of drugs which would decrease magnesium level by increasing its renal excretion such as ethanol, cyclosporine, cisplatin, aminoglycosides, amphotericin B, capreomycin, diuretics, furosemide, ethacrynic acid, acetazolamide, thiazides and chlorothalidone were main exclusion criteria.

Besides, 50 healthy subjects without any family history or evidences of migraine matched for inclusion and exclusion criteria were randomly selected from hospital personnel as the control group. The study protocol was approved by the Ethics Committee of the Semnan University of Medical Sciences and all patients gave their consent to participate this study.

Baseline data were collected by face to face interviewing or from the hospital recorded files, including demographics, medical and drug history, clinical manifestations and baseline laboratory parameters. Venous blood sample were drawn from the antecubital vein using a disposable injector into silicon-coated plastic tubes with EDTA-K and centrifuged at 3000 rmp for 10 minutes. These blood samples were collected between 8 a.m. and 10 a.m. after an overnight fast and were sent to the hospitals laboratories for measuring serum Mg level. Serum Mg level was measured by xylidyl blue method using Pars Azmoon Company kits, Iran.

### Data analysis

Results were reported as mean ± standard deviation (SD) for the quantitative variables and percentages for the categorical variables. The groups were compared using the t-test for the continuous variables and the chi-square test (or Fisher′s exact test if required) for the categorical variables. P values of 0.05 or less were considered statistically significant. All the statistical analyses were performed using SPSS version 13.0 (SPSS Inc., Chicago, IL, USA) for Windows.

## Results

Fifty patients with migraine headache and 50 individuals without this events or its history were eligible for study participation. No significant differences across the two groups were noted regarding sex ratio (female to male ratio in both group was 6.1) and mean age 28.94 ± 7.23 versus 30.00 ± 7.34 years, p = 0.468). Thirty one patients in the diseased group (62.0%) and 29 subjects in the control group (58.0%) were younger than 30 years old. In the group with migraine headache, no significant difference was found in the serum total Mg levels within and between migraine headache attacks (1.86 ± 0.41 mg/dl versus 1.95 ± 0.35 mg/dl, p = 0.224). But, serum total Mg level was notably lower in the group with these attacks compared to the control group (1.86 ± 0.41 mg/dl versus 2.10 ± 0.23 mg/dl, p < 0.001).

## Discussion

The current study emphasize on the lower level of serum Mg in migraineurs in comparison with the healthy group with the mean differences of 0.24 mg/dl. Some studies revealed similar findings. Sarchielli and his colleagues [6] showed that the migraine sufferers with and without aura and tension-type headache had significantly lower levels of serum and salivary magnesium concentrations in the interical periods than a group of healthy young individuals.

In their study, serum magnesium levels tended to be further reduced during attacks in all patient groups studied. They concluded that serum magnesium levels and to a lesser extent salivary magnesium levels might express indirectly the lowering of brain extracellular magnesium concentration which occurs in migraine patients both in the intererictal periods and ictally. Some studies have also shown that patients with cluster headaches and classic or common migraine have low levels of magnesium [7]. It has been suggested that magnesium might be involved in migraine pathogenesis by counteracting vasospasm, inhibiting platelet aggregation, and the stabilization of cell membranes [8]. In addition, magnesium also plays a role in the control of vascular tone and reactivity to endogenous hormones and neurotransmitters, through its relationship with the NMDA receptor [9]. It has been also revealed that migraineurs have low brain magnesium during migraine attacks and may also have a systemic magnesium deficiency [10].

## Conclusion

Based on our result and according to the findings obtained in similar researches, assessing serum Mg level might predict migraine attacks and its symptoms as well as might help physicians to determine optimal dose of administered Mg for achieving appropriate therapeutic outcome in these patients. Hence, its diagnostic and therapeutic role in migraineurs should more assess.
